# Invoking self-related and social thoughts impacts online information sharing

**DOI:** 10.1093/scan/nsad013

**Published:** 2023-03-03

**Authors:** Christin Scholz, Elisa C. Baek, Emily B Falk

**Affiliations:** Amsterdam School of Communication Research, University of Amsterdam, Amsterdam 1018WV, The Netherlands; Department of Psychology, University of California, Los Angeles, Los Angeles, CA 90095, USA; Annenberg School of Communication, University of Pennsylvania, Philadelphia, PA 19104, USA; Department of Psychology, University of Pennsylvania, Philadelphia, PA 19104, USA; Wharton Marketing Department, University of Pennsylvania, Philadelphia, PA 19104, USA

**Keywords:** information sharing, sharing goals, causal manipulation, fMRI, mechanisms

## Abstract

Online sharing impacts which information is widely available and influential in society. Yet, systematically influencing sharing behavior remains difficult. Past research highlights two factors associated with sharing: the social and self-relevance of the to-be-shared content. Based on this prior neuroimaging work and theory, we developed a manipulation in the form of short prompts that are attached to media content (here health news articles). These prompts encourage readers to think about how sharing the content may help them to fulfill motivations to present themselves positively (self-relevance) or connect positively to others (social relevance). Fifty-three young adults completed this pre-registered experiment while undergoing functional magnetic resonance imaging. Ninety-six health news articles were randomly assigned to three within-subject conditions that encouraged self-related or social thinking or a control. Invoking self-related or social thoughts about health-related news (*vs* control) (i) causally increased brain activity in a priori regions of interest chosen for their roles in processing social and self-relevance and (ii) causally impacted self-reported sharing intentions. This study provides evidence corroborating prior reverse inferences regarding the neural correlates of sharing. It further highlights the feasibility and utility of targeting neuropsychological processes to systematically facilitate online information spread.

Information sharing within online social networks critically affects which content is widely available, frequently viewed and impactful (Jeong and Bae, 2017; Scharkow *et al.*, 2020). Diverse actors from public health officials (Slavik *et al.*, 2021), to marketers (Taecharungroj, 2017), to everyday social media users (Lindström *et al.*, 2021) assign a high value to the number of shares their content receives and have a strong interest in increasing that number. Yet, purposefully impacting sharing behavior online remains difficult. Recent work has employed functional neuroimaging to identify neuropsychological drivers of sharing decisions (Scholz *et al.*, 2017). This research in conjunction with theorizing in marketing, psychology (Berger, 2014), communication science (Cappella *et al.*, 2015) and neuroscience (e.g. Bartra *et al.*, 2013) has informed a value-based theory of sharing. This theory suggests that the content is more likely to be shared if the act of sharing is perceived to be valuable. In turn, the perceived value depends on whether would-be sharers believe that sharing a piece of content would fulfill key psychological motivations, especially motives to present oneself positively (self-relevance motives) and relate positively to others (social relevance motives; Scholz *et al.*, 2020a). It has been proposed, but never tested, that this theory can inform a novel, mechanistic intervention approach that systematically impacts sharing by supporting would-be sharers in identifying opportunities to fulfill their sharing motives. In this pre-registered neuroimaging experiment (https://osf.io/n9vpz), we tested whether helping participants to fulfill self- and social relevance motives by sharing health news articles would increase the perceived value of sharing and, ultimately, sharing intentions. This study provides a strong test of prior theorizing and demonstrates the feasibility of approaches that target neuropsychological processes to facilitate online information spread.

## A mechanism-driven approach to encourage sharing

Extant sharing research frequently studied which content characteristics encourage sharing without explaining the mechanisms of these relationships. For instance, is emotional content shared often (Stieglitz and Dang-Xuan, 2013; Wang *et al.*, 2017) because experiencing emotions motivates would-be sharers to act or because would-be sharers expect more ‘Likes’ for the shared content if receivers are emotional? Given this uncertainty about mechanisms, content-focused insights primarily inform content-focused interventions, which involve labor-intensive editing of individual pieces of content to influence sharing. This is impractical, especially for content that does not naturally have ‘shareable’ characteristics and/or pursues goals other than social popularity (e.g. inform or persuade) that could be compromised by sharing-focused editing.

Instead, a sharer-focused, mechanism-driven approach, like the one tested here, asks why people share content and supports sharers in identifying opportunities to use content sharing to fulfill these sharing motives. This approach is easily scalable across pieces and types of content because it guides how would-be sharers think about the content rather than modifying individual pieces of content.

## Self- and social relevance and the perceived value of sharing

Which psychological drivers of sharing should be targeted? Prior work relying on self-report produced important insights (Berger, 2014; Cappella *et al.*, 2015) but remains limited by social desirability and memory biases associated with post-hoc reporting. Interdisciplinary interest in sharing behavior has also generated incomparable measurement scales of sharing motives (Marwick and Boyd, 2011; Lee and Ma, 2012; Kim *et al.*, 2021), which makes it difficult to draw generalizable conclusions and give consistent advice to content creators.

Functional neuroimaging has proven useful in supplementing self-reports to predict real-world behaviors (Falk *et al.*, 2012; Genevsky and Knutson, 2015; Jai *et al.*, 2021) and understand psychological mechanisms (Vezich *et al.*, 2017; Scholz *et al.*, 2020b; Chan *et al.*, 2021; Casado-Aranda *et al.*, 2022). Neuroimaging provides unobtrusive, real-time access to neuropsychological mechanisms underlying sharing decisions. In addition, by revealing the physiological architecture underlying behavior, neuroimaging helps distinguish physiologically plausible from implausible theories of behavior (Huskey *et al.*, 2020). Finally, neuroimaging can identify common concepts underlying otherwise incomparable, context-specific self-report measures (Lieberman, 2010) like those created for sharing motives.

In the context of online sharing, neuroimaging studies found robust correlations between brain activity during content exposure and sharing intentions in study participants (Baek *et al.*, 2017) and real sharing decisions in large online populations (Scholz *et al.*, 2017; Motoki *et al.*, 2020). Regions in which activity correlates with sharing include clusters within medial prefrontal cortex, striatum, precuneus, temporoparietal junction and temporal lobes. Prior work concluded that these patterns of activity may be indicative of value-related, social and self-related processing (Meshi *et al.*, 2015; Baek *et al.*, 2017).

Integrating these correlational neuroimaging studies with the existing theorizing about brain function, sharing and motivation psychology informed the value-based model of sharing (Scholz *et al.*, 2020a). Neuroscientists have documented robust relationships between activity in the brain’s value system (consisting of, among others, clusters in ventral striatum and ventromedial prefrontal cortex) and choice behavior across contexts (Bartra *et al.*, 2013). To inform decisions, the value system computes the subjective value of choice options (e.g. whether or not to share) by summarizing and weighting diverse decision attributes (e.g. perceived self- and social relevance), which may be encoded elsewhere in the brain (Levy and Glimcher, 2012).

To determine whether sharing is a good idea (‘valuable’), a would-be sharer may consider, among others, whether sharing reflects positively on them and improves/maintains their social relationships. This is consistent with the work in motivation psychology and the work on sharing in communication science and marketing. Motivations to connect positively with others (Baumeister and Leary, 1995) and to hold and present a positive image of ourselves (Mezulis *et al.*, 2004) inform human behaviors across virtually all aspects of life. Furthermore, broad concepts of self- and social relevance may be described as the ‘smallest common denominators’ of numerous context-specific concepts that are measured using context-specific self-reports by sharing researchers in different disciplines (Scholz *et al.*, 2017). For instance, social relevance considerations may manifest as thoughts about whether sharing leads to good conversations or a fight, and self-relevance considerations may include thoughts about whether sharing makes the sharer look smart or whether it represents their values. As such, the central role of self-related and social thought processes proposed by the value-based model of sharing is consistent with prior interdisciplinary theorizing and empirical data and provides a physiologically plausible account of sharing decisions.

The current study makes two important contributions. First, neuroimaging work shows that simple tasks that trigger self-related, social and value-related thoughts (e.g. evaluating whether personality traits describe ‘me’ or indicating willingness to pay for some stimulus) engage regions in which activity also correlates with sharing (e.g. Murray *et al.*, 2012; Bartra *et al.*, 2013; Dufour *et al.*, 2013). Yet, each of these brain regions is also involved in other thought processes (Poldrack, 2006). First, we aim to strengthen prior reverse inferences about the psychological mechanisms underlying the neural correlates of sharing decisions (i.e. the value-based model of sharing) by manipulating how would-be sharers think about to-be-shared content (encouraging self-related and social thoughts) and observing the resulting activity in a priori brain regions of interest (ROIs), chosen for their role in self-related, social and value-related processing. Second, we test causal manipulation effects on sharing intentions to examine the practical utility of neuroscientifically informed, mechanistic interventions.

This pre-registered study (https://osf.io/n9vpz) tests the following hypotheses:

There will be a main effect of our mechanism-driven manipulation targeting how would-be sharers think about to-be-shared content on neural activity in brain ROIs that are meta-analytically associated with self-related, social and value-related cognition so that neural activity will be higher in

- the social relevance *vs* control condition, and
- the self-relevance *vs* control condition.
- Research question: are there differences in ROI activity or whole-brain activity when comparing the self- and social relevance conditions?

Self-reported sharing likelihood and perceived benefits of sharing will be higher in

- the social relevance *vs* control condition, and
- the social relevance *vs* control condition.
- Research question: are there differences in sharing likelihood and perceived benefits of sharing between the self- and social relevance conditions?

## Interconnections between social, self- and value-related thought

We chose not to pose directional hypotheses regarding differences between the effects of manipulations targeting self-related and social thought processes. This is partially motivated by a strong conceptual and empirical overlap between social, self- and value-related processing. Theorizing about the ‘social self’ (Bretherton, 1991) posits that our very sense of self is socially determined because definitions of who we are are often based on which social groups we are/are not part of. In the context of sharing, presenting oneself positively (supporting self-related motives) likely requires social thought to determine what others perceive as ‘positive’. In turn, relating positively to others likely requires self-related thought to identify characteristics of the self that are relevant to others (Scholz *et al.*, 2020b). Furthermore, prior work suggests that the concepts of value and self are strongly interconnected and, in fact, barely distinguishable (D’Argembeau *et al.*, 2012). This connection is evident in universal self-serving biases that lead us to assign a higher value to entities and activities if we perceive them as self-relevant (Mezulis *et al.*, 2004). The conceptual overlap between social, value- and self-related processing means that these processes are hardly distinguishable using neuroimaging data without applying highly specialized study designs and analysis techniques focused on this purpose (e.g. Parelman *et al.*, 2022). This is beyond the scope of this manuscript.

## Methods

**Fig. 1.**
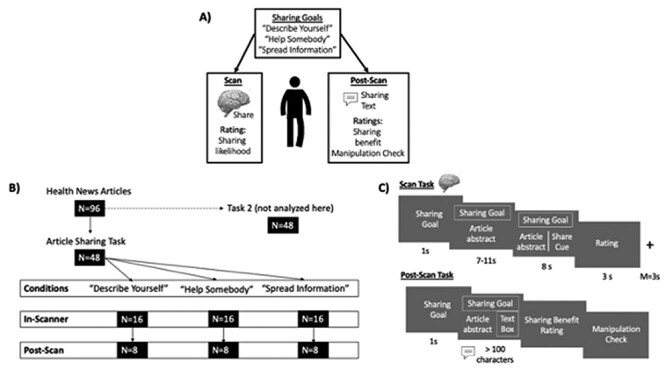
(A) The data overview. (B) Random assignment of *N* articles to condition for each participant in the scan and post-scan tasks. Arrows represent random sub-selection of articles from the preceding sample. (C) Scan- and post-scan task schemata and timing. The post-scan task was self-paced and required participants to write sharing texts (minimum 100 characters).

We conducted a pre-registered within-subject experiment in young adults. First, during a functional magnetic resonance imaging (fMRI) session, participants considered sharing headlines and abstracts of New York Times articles following one of the three experimentally manipulated goals (‘Describe Yourself’, ‘Help Somebody’ and ‘Spread Information’; Figure 1A) before indicating their likelihood to share each article. Next, during a post-scan period, participants composed texts that they might use to share each article (again following one of the three goals) and rated the extent to which they thought sharing would be beneficial. Manipulation check items (described later) were also assessed after the scan. All participants further completed another task that is not analyzed here (The task order was randomized.) and an online survey including demographic and individual difference measures. Here, we only analyze survey measures regarding participants’ interest levels and consumption habits regarding health news, their level of enjoyment of conversations about health and their age and gender. All manipulations and pre-registered analyses concerning the hypotheses described previously are reported in this manuscript.^1^ All participants provided informed consent, and study procedures received ethical approval at the University of Pennsylvania.

### Participants

Participants were recruited through convenience sampling using on-campus recruitment tools and Facebook ads. Eligible participants did not have counter-indications for fMRI scanning (see Supplementary Materials) and were native English speakers. In total, 53 participants completed the tasks. The sample size was determined a priori based on the availability of funding and resources and pre-registered before the data analysis (https://osf.io/n9vpz). Three participants were excluded from all analyses because they were found to be ineligible after obtaining consent (*N* = 2) or terminated participation early (*N* = 1). Fifty eligible participants were included in at least a subset of analyses. They were between 18 and 35 years old (*M* = 23.02, s.d. = 4.32) and predominantly female (68.00% female and 32.00% male). All partial data exclusions and additional participant characteristics are described in the Supplementary Materials.

### Articles

Ninety-six health news articles published on the New York Times website served as stimuli. Articles were sub-selected from a census of health-related articles published between July 2012 and February 2013 (Kim, 2015). Articles were chosen based on a shared topic (healthy living and physical activity) by applying a keyword search and limiting the word count range (*M* = 28.42 words; s.d. = 4.53; range = 17–35). For each participant, a random selection of 48 articles was assigned to the Article Sharing Task. The remaining 48 articles were used for the second task that is not analyzed here. Articles assigned to the Article Sharing Task were randomly allocated to the three within-subject conditions per participant, so that articles differed in their condition assignment between participants. Each participant viewed 16 articles per condition in the scan task (total *N* = 48) and a random subset of eight articles per condition in the post-scan task to manage participant burden (total *N* = 24; see Figure 1B).

### Within-subject manipulation

The two experimental prompts encouraged participants to identify opportunities for using a piece of content to relate positively to others (use the article to ‘Help Somebody’; increasing social relevance) or present themselves positively (use the article to ‘Describe Yourself’; self-relevance). In the control condition, participants were asked to objectively ‘Spread Information’ given in the article and thereby did not receive help in identifying opportunities to fulfill their sharing motives. Supplementary Table S1 prints task instructions and example texts participants generated in the post-scan task.

### Article Sharing fMRI Task

The Article Sharing Task that participants completed inside the fMRI scanner (Figure 1C) consisted of two runs (24 trials each). Per trial, participants were first shown a condition cue (1 s) indicating the current sharing goal (‘Describe Yourself’, ‘Help Somebody’ or ‘Spread Information’), followed by a reading period during which participants read an article’s headline and abstract (7–11 s, depending on the article length). During a subsequent sharing period, participants were prompted to consider what they might say or write to another study participant if they were to share the article with them, keeping in mind their current sharing goal (8 s). The sharing goal remained visible on the screen throughout the reading and sharing periods. Finally, participants had three seconds to rate their likelihood to share the article in real life (3 s; 1: very unlikely and 5: very likely). Trials were separated by a jittered fixation period (*M* = 3 s).

### Post-scan task

The post-scan task (Figure 1C) consisted of 24 trials (8/condition). Per trial, participants were first informed about the current condition and then re-read one article, which they had already seen in the scan task, before writing a short text (minimum 100 characters) that they might use to share the article with another study participant, keeping in mind their current sharing goal. Subsequently, participants were asked to rate whether it would be beneficial for them to share the article with somebody else (1: very unlikely and 5: very likely). In addition, participants provided three ratings that served as manipulation checks. Specifically, they rated the extent to which they thought that the message they just wrote successfully fulfilled each of the three sharing goals (‘Describe Yourself’, ‘Help Somebody’ and ‘Spread Information’; 1/very unlikely and 5/very likely).

### Brain ROIs

**Fig. 2.**
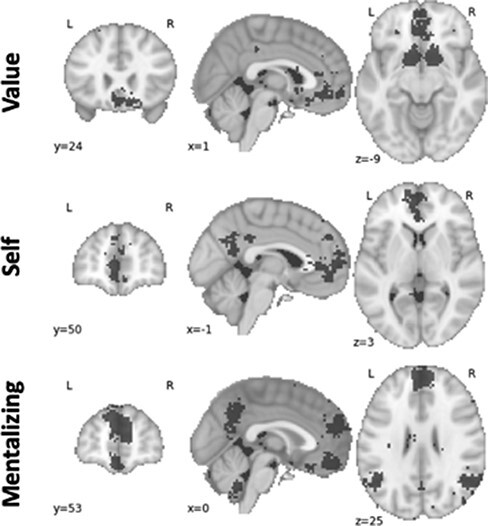
Regions of interest extracted from www.Neurosynth.org.

As pre-registered (https://osf.io/n9vpz), we defined a priori brain ROIs using Neurosynth, a tool that conducts automatic meta-analyses of the existing neuroimaging literature (Yarkoni *et al.*, 2011). Specifically, using association test masks, we chose ROIs in which brain activity is meta-analytically associated (False Discovery Rate, *P* < 0.01) with the terms ‘mentalizing’ (i.e. thinking about the thoughts of others; Frith and Frith, 2006), ‘self’ and ‘value’ (Figure 2). We focused on mentalizing as a special type of social processing, following prior work on the neural correlates of sharing (Baek *et al.*, 2017) as well as theorizing, suggesting the importance of considering the thoughts of others in sharing decisions (Scholz *et al.*, 2020b; Baek *et al.*, 2020).

In addition, the Supplementary Materials detail the definition of four additional ROIs used in exploratory analyses aiming to determine the specificity of our results to our a priori ROIs and the distinguishability of condition effects on activity in individual ROIs after the overlap was removed.

To quantify the extent to which an ROI was engaged by a given experimental condition, we first estimated voxel-wise condition effects on brain activity and then averaged relevant parameter estimates across all voxels within the ROI mask (detailed later).

### fMRI data acquisition and pre-processing

We collected two runs of functional images while participants completed the Article Sharing Task and structural and T1- and T2-weighted images using a 3-Tesla Prisma Siemens scanner. Scanning sequences are described in detail in the Supplementary Materials. We pre-processed fMRI data using fmriprep (Esteban *et al.*, 2017) with the Freesurfer reconstruction option. We normalized structural T1- and T2-weighted images to the skull-stripped MNI template (‘MNI152_T1_1mm_brain.nii’) and segmented normalized images into gray matter, white matter and cerebrospinal fluid. Afterwards, the surfaces of the cortical sheet were reconstructed from the T1- and T2-weighted images. Functional images were spatially realigned and co-registered to the structural images and then smoothed using a 6 mm Gaussian kernel.

### Neuroimaging data analyses

Pre-processed fMRI data were analyzed using the general linear model implemented in Statistical Parametric Mapping (SPM) software (SPM12, Wellcome Department of Cognitive Neurology, Institute of Neurology, London, UK). The first-level model included three regressors modeling cue screens pooled by condition (one regressor each for ‘Describe Yourself’, ‘Help Somebody’ and ‘Spread Information’ trials), three regressors modeling the entire period during which the article was on the screen (read and share screens) pooled by condition and one regressor for rating screens. In addition, we included a regressor of no-interest pooling trials with missing ratings and nine nuisance motion regressors. A high-pass filter was applied to remove low-frequency noise (128 s).

We computed first-level contrasts for all pair-wise condition comparisons for trial periods during which articles were visible on screen. For each ROI and each participant, we then averaged the resulting voxel-wise parameter estimates for all voxels within a given ROI (yielding one number per person, per ROI) using functions provided by Numpy (Version 1.20.3, Harris *et al.*, 2020) and Nilearn (Version 0.7.1, Abraham *et al.*, 2014). To determine whether activity in a given ROI differed significantly between conditions, we conducted one-sample *t*-tests per contrast. At a conventional alpha level of 0.05, these tests have 79% power to detect medium effect sizes of Cohen’s *d* = 0.35, according to a post-hoc power analysis using G*Power (Faul *et al.*, 2007).

For exploratory multilevel modeling of indirect effects, we estimated an additional first-level model including separate regressors per trial to extract brain activity in response to each article (i.e. a beta-series model producing one value per article, per participant). Each regressor included the entire period within the trial during which the article was visible on screen. We then conducted exploratory multilevel regression analysis using indirect.mlm in R (Page-Gould & Sharples, 2016) to test the indirect effects of condition on sharing likelihood ratings through brain activity in the three ROIs (see details in Supplementary Materials) and effects of condition on activity in the value-related ROI, mediated by activity in the other pre-registered ROIs.

### Analysis of ratings

We used multilevel models to analyze rating data using R lmerTest (Kuznetsova *et al.*, 2019). Specifically, we regressed sharing likelihood or benefit of sharing ratings, respectively, on a trial condition factor and included random intercept terms for participants and article ID to account for nonindependence due to repeated observations per person and article. Based on a simR (Green and MacLeod, 2016) post-hoc power analysis with 1000 simulations and an alpha level of 0.05, these tests have 78.2% power (95% Confidence Interval (CI) [75.51–80.82%]) to detect a small effect size of 0.15. Small effect sizes are relevant in the context of sharing intentions, given that the size of online audiences may lead to changes in large groups of people even if only a small proportion of a population is affected by an intervention. We deviated from our pre-registered analysis plan for the analysis of ratings because the repeated-measures data are more appropriately analyzed using multilevel models. The results of the pre-registered analysis support substantially similar conclusions and are reported in the Supplementary Materials. The same procedure was applied for manipulation check ratings.

## Results

### Manipulation checks

First, we tested whether participants perceived themselves to be successful in distinguishing between conditions. Specifically, in the post-scan task, participants rated each message they composed on the likelihood that the message would achieve each of the three goals (‘Help Somebody’, ‘Describe Yourself’ and ‘Spread Information’; only one of which was assigned through instructions for the current trial). As expected (see Hypothesis 1.1 in https://osf.io/n9vpz), we found that for all conditions, participants perceived their messages to be more successful in fulfilling a given goal when it was assigned by condition instructions than when other goals were assigned (Table 1; top half).

**Table 1.** Manipulation check results

Conditions	Help Somebody	Describe Yourself	Spread Information
Help Somebody		1.03 [0.89–1.16]	0.87 [0.73–1.00]
Describe Yourself	1.63 [1.48–1.78]		1.80 [1.65–1.95]
Spread Information	0.46 [0.33–0.59]	0.74 [0.61–0.86]	

*Notes*: For all three goals, participants reported that their sharing texts achieved a given goal more effectively when it was assigned via condition prompts than when it was not. This table shows beta estimates and 95% CIs derived using multilevel models regressing self-reported goal fulfillment for each goal (rows) on condition factors, such that column condition > row condition. All *P*-values <0.001.

Second, in an exploratory analysis, we examined condition effects on the language participants used while sharing articles in the post-scan task using LIWC 2007 dictionary (Pennebaker *et al.*, 2007). This language analysis provided evidence that participants executed the task as instructed. Communicators used first-person singular forms (category: ‘i’) most often in the ‘Describe Yourself’ condition. Social language (category: ‘social’) was used most often in the ‘Help Somebody’ condition to link articles with other people or give advice, and health-related language (category: ‘health’) was used most frequently in the ‘Spread Information’ condition. Both manipulation checks largely replicate results from a pre-test conducted using the same articles and manipulations in a sample of online participants. Detailed information about the pre-test and language analyses are presented in the Supplementary Materials.

### Causal effects of sharing goals on brain activity

**Fig. 3.**
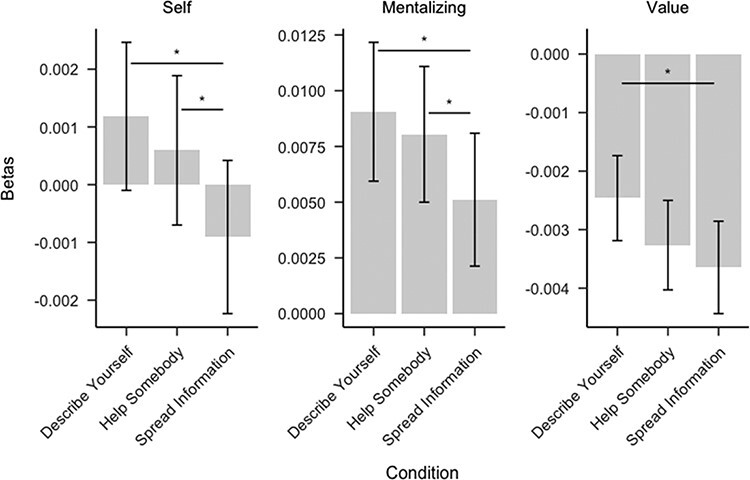
The condition effects on brain responses per condition (*vs* rest). Error bars represent standard errors. Significance indicators are based on one-sample *t*-tests evaluating average differences in brain activity between condition pairs. *P < 0.05.

Next, we tested whether sharing goals causally affected brain activity in ROIs chosen for their involvement in mentalizing, self- and value-related processing (Figure 3). As expected (see Hypothesis 2.1 in https://osf.io/n9vpz), we found increased activity in the mentalizing (*M* = 0.004,  95% CI $\left[ {0.001,0.007} \right]$, $t\left( {41} \right) = 2.42$, *P* = 0.020), self-related ($M = 0.002$, 95% CI $\left[ {0.001,0.004} \right]$, $t\left( {41} \right) = 2.68$, *P* = 0.011) and value-related ($M = 0.001$, 95% CI $\left[ {0.000,0.002} \right]$, *t*(41) = 2.22, *P* = 0.032) ROIs in the ‘Describe Yourself’ compared to the ‘Spread Information’ condition. The ‘Help Somebody’ (*vs* ‘Spread Information’) condition also yielded greater activity in the mentalizing (*M* = 0.003, 95% CI $\left[ {0.000,0.006} \right]$, $t\left( {41} \right) = 2.20$, *P* = 0.034) and self-related ($M = 0.002$, 95% CI $\left[ {0.000,0.003} \right]$, $t\left( {41} \right) = 2.52$, *P* = 0.016) processing ROIs but not directly in the value ROI. However, exploratory tests of the indirect effect of condition on value-related ROI activity through neural activation in both self-related (B = 0.124, 95% CI [0.029, 0.221]) and mentalizing (B = 0.126, 95% CI [0.038, 0.219]) ROIs were significant (see Supplementary Materials for details).

ROI activity did not differ between the ‘Describe Yourself’ and ‘Help Somebody’ conditions (see research question 2.1c in https://osf.io/n9vpz; Figure 3). In an additional exploratory analysis, we replicated these results using an alternative set of non-overlapping ROIs chosen to operationalize mentalizing, self- and value-related processing, providing some additional evidence that our manipulations uniquely engaged each type of cognition (see Supplementary Materials). Finally, exploratory ROI and planned whole-brain analyses did not show widespread condition effects outside the a priori ROIs (see Supplementary Materials).

### Causal effects of sharing goals on sharing likelihood

**Fig. 4.**
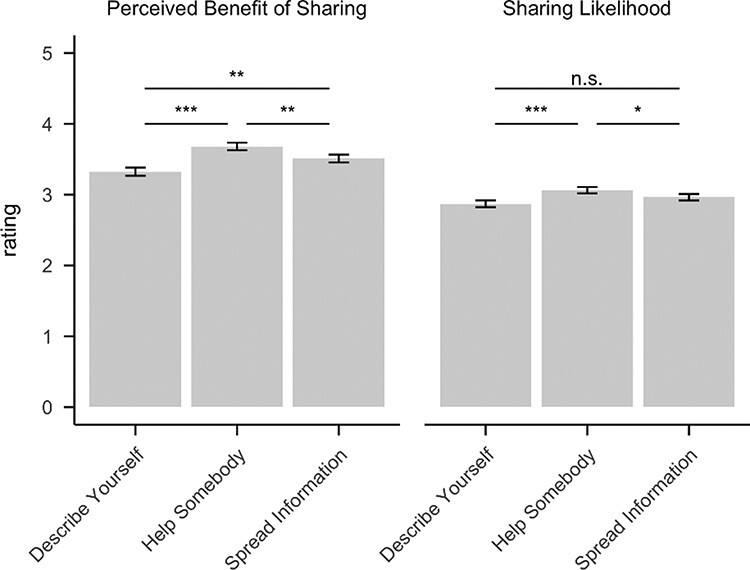
The condition effects on ratings. Error bars represent standard errors. Significance indicators are based on the regression models reported in Table 2. *P < 0.05, ** *P* < 0.01, *** *P* < .001.

Next, we tested whether the manipulation causally impacted self-reported sharing likelihood and perceived benefits of sharing (see Hypothesis 3.1 a–c in https://osf.io/n9vpz). As hypothesized, multilevel models indicate that participants provided higher ratings on perceived benefits of sharing and sharing likelihood in the ‘Help Somebody’ (*vs* ‘Spread Information’) condition. Unexpectedly, participants reported that sharing would be less beneficial and were directionally, yet not significantly, less likely to report wanting to share articles in the ‘Describe Yourself’ (*vs* ‘Spread Information’) condition. Both perceived benefits of sharing and sharing likelihood were lower in the ‘Describe Yourself’ compared to the ‘Help Somebody’ condition (Table 2 and Figure 4).

**Table 2.** Condition effects on communicator ratings [B (SE), *P*-value]

Rating	Describe Yourself > Spread Information	Help Somebody > Spread Information	Describe Yourself > Help Somebody
Sharing likelihood	−0.07 (0.06), *P* = 0.203	0.13 (0.06), *P* = 0.024	−0.20 (0.06), *P* < 0.001
Benefit of sharing	−0.20 (0.06), *P* = 0.001	0.18 (0.06), *P* = 0.006	−0.38 (0.06), *P* < 0.001

To further investigate the unexpected effects of the ‘Describe Yourself’ condition, , we ran an exploratory trial-wise, multilevel regression analysis to test potential indirect effects of condition (‘Describe Yourself’ > ‘Spread Information’) on sharing ratings entering the three ROIs as mediators. All three indirect effects were positive and statistically significant, suggesting that there are two mechanisms that explain the association between ‘Describe Yourself’ instructions (*vs* control) and sharing likelihood ratings: a direct effect indicating lower sharing likelihood ratings for ‘Describe Yourself’ instructions and an additional indirect effect where ‘Describe Yourself’ instructions have a positive effect on sharing likelihood ratings via increases in the activation of specific ROIs (see Supplementary Materials for details).

## Discussion

The value-based theory of sharing posits that sharing content online is a value-based decision and that self- and social relevance of the to-be-shared content contributes to the perceived value of sharing (Scholz *et al.*, 2020a). We show that helping would-be sharers to identify opportunities to fulfill their self-related and social sharing motives leads to increased brain activity in regions that were implicated in sharing decisions in correlational neuroimaging work, as well as changes in self-reported sharing intentions. Our results highlight the potential utility of interventions that target neuropsychological mechanisms underlying sharing to systematically impact sharing behavior without requiring labor-intensive and potentially disruptive edits to content characteristics (e.g. directly editing news headlines).

First, our sharing goal manipulation causally impacted brain activity in hypothesized ROIs while participants considered sharing health news articles. We further provide evidence that brain activity outside these ROIs was not significantly increased by the experimental conditions. Consistent with pre-registered hypotheses, activity in ROIs associated with self-related, social (specifically mentalizing) and value-related processing was higher when participants used articles to ‘Describe Yourself’ compared to ‘Spread Information’. This is consistent with the idea that people value positive social interaction (Tamir and Hughes, 2018) like sharing (especially self-related) information with others (Tamir and Mitchell, 2012; Tamir *et al.*, 2015) and derive value from self- and social relevance motives (Scholz *et al.*, 2020a). To describe themselves, participants likely considered their own characteristics/experiences (self-related processing) in relation to interests of the audience they may share with (social processing). In line with prior theorizing (Scholz *et al.*, 2020a), we propose that ‘Describe Yourself’ instructions aided participants in identifying opportunities to fulfill key motivations (e.g. to present themselves positively) by sharing the article, which increased the perceived value of sharing. Self-related and social but not value-related activity was further stronger in ‘Help Somebody’ compared to control trials. To ‘Help Somebody’ using an article, communicators likely considered the potential needs and interests of their receivers in relation to their own expertise.

The non-significant effect of the ‘Help Somebody’ condition on value-related processing conflicts with prior work that emphasizes the high value humans place on positive social connections (Baumeister and Leary, 1995) and information sharing as a means of fulfilling this motive (Berger, 2014). Our study may underestimate the effect of ‘Help Somebody’ instructions on activity in the value of ROI because our participants shared with strangers, which may be less valuable than sharing to help friends. Alternatively, our manipulation may affect value-related brain activity indirectly as suggested by value-based decision-making theory. Indeed, we found significant indirect effects of condition (‘Help Somebody’ > ‘Spread Information’) on activity in the value of ROI through activity in both the self-related and social processing ROIs. However, our cross-sectional neural measurements preclude causal or strong directional claims.

Finally, the results of one prior study examining sharing decisions for video advertisements (Motoki *et al.*, 2020) differed from work on health news sharing (e.g. Scholz *et al.*, 2017) in that it did not find significant relationships between value-related brain activity and sharing. Although this relationship was significant in the current study (Supplementary Table S9), we encourage more research on when value-related brain activity is associated with sharing and how it can be induced systematically.

Although there were some linguistic distinctions in how participants shared content across conditions (see exploratory analysis in Supplementary Figure S1), we did not hypothesize and did not find differences in ROI activity between the ‘Describe Yourself’ and ‘Help Somebody’ conditions. This is in line with a strong theoretical and empirical overlap between social and self-related (Bretherton, 1991) and between value- and self-related (Enzi *et al.*, 2009) thought processes. Exploratory analyses using non-overlapping ROIs suggest that the manipulations led to changes in cognitions that are uniquely represented by each ROI rather than a single, common underlying component. Yet, even in non-overlapping ROIs, activity was highly correlated and is, thus, not fully distinguishable. Clear distinctions between social, self- and value-related processing usually require complex, dedicated study designs and analyses (e.g. Enzi *et al.*, 2009; Parelman *et al.*, 2022). We argue that this limited specificity is a feature of the natural cognitions underlying sharing decisions. For instance, self-related concerns (e.g. presenting oneself positively) require social thoughts (e.g. what do others consider ‘positive’?) and vice versa (Scholz *et al.*, 2020b). At this stage, our work highlights the utility of both ‘Help Somebody’ and ‘Describe Yourself’ instructions but does not clearly distinguish between them.

In sum, our neuroimaging and text data support pre-registered hypotheses, suggesting that inducing self-related and social thought processes increases brain activity in regions associated with self-related, social and value-related thoughts during sharing decisions. This extends prior work that relied on reverse inferences to interpret neural correlates of sharing. Yet, some limitations remain with regard to these conclusions. For instance, future work may increase specificity in distinguishing neural responses to different manipulation strategies and describing the precise cognitions that underlie ROI activity (e.g. the value of ROI may operationalize separate yet related concepts like value and reward).

Second, we found condition effects on sharing intentions and perceived benefits of sharing. As expected, ‘Help Somebody’ (*vs* ‘Spread Information’) instructions increased both ratings, supporting prior theorizing on the role of social processing in sharing decisions (Falk and Scholz, 2018). In contrast, ‘Describe Yourself’ instructions decreased both ratings compared to the ‘Help Somebody’ and control conditions. This conflicts with prior work that highlighted the importance of motives like self-presentation and self-enhancement in sharing (e.g. (Barasch and Berger, 2014; Bazarova and Choi, 2014) and demonstrated that opportunities to share self-related information are perceived as valuable (Tamir and Mitchell, 2012). One possible explanation is social desirability. Although participants may have perceived sharing self-related information as inherently valuable, as indicated by our neuroimaging data, explicitly admitting this through self-report may feel non-normative/narcissistic. Consistent with this hypothesis, exploratory analyses showed a positive indirect effect of ‘Describe Yourself’ (*vs* ‘Spread Information’) instructions on sharing likelihood ratings through brain activity in regions associated with self-related, social and value-related processing. Our cross-sectional data preclude strong claims about causality. Nevertheless, while self-report data suggested that the ‘Describe Yourself’ condition is ineffective or even harmful, our multimethodological approach underlines the added value of neuroimaging data, which suggests that this manipulation may, after all, have intended effects and requires further investigation. For instance, positive effects may be expected on passively logged sharing behavior that is unaffected by social desirability concerns, on sharing intentions targeting friends rather than strangers, or in response to more subtle manipulations.

In sum, instructions to share health news articles to ‘Describe Yourself’ and ‘Help Somebody’ significantly increased activity in brain regions meta-analytically associated with self-related, social and value-related processing. This validates prior reverse inferences following correlational work on the role of these cognitions in sharing decisions (Falk and Scholz, 2018). Especially, ‘Help Somebody’ instructions further causally affected self-report measures of sharing intentions. As such, a neuroscience-inspired, scalable manipulation of how would-be sharers think about online content may help enhance the spread of diverse information online. Next, we encourage additional work to explicitly test the generalizability of these effects outside the lab (IJzerman *et al.*, 2020).

## Supplementary Material

nsad013_Supp

## Data Availability

The pre-registration (https://osf.io/n9vpz), data and code needed to reproduce all analyses presented in this manuscript and task stimuli (https://osf.io/pxnmw/) are available on our project page on the Open Science Framework (OSF) website (DOI: 10.17605/OSF.IO/PXNMW).

## Footnotes

1 The pre-registration includes one additional research question that is beyond the scope of the current manuscript.

## Citation diversity statement

Recent work in several fields of science has identified a bias in citation practices such that papers from women and other minority scholars are undercited relative to the number of such papers in the field. Here, we sought to proactively evaluate whether references reflect the diversity of the field in thought, form of contribution, gender, race, ethnicity and other factors. First, we obtained the predicted gender of the first and last author of each reference by using databases that store the probability of a first name being carried by a woman. By this measure (and excluding self-citations to the first and last authors of our current paper), our references contain 8.89% woman(first)/woman(last), 13.33% man/woman, 14.17% woman/man and 63.6% man/man. This method is limited in that (i) names, pronouns and social media profiles used to construct the databases may not, in every case, be indicative of gender identity, and (ii) it cannot account for intersex, non-binary or transgender people. Second, we obtained the predicted racial/ethnic category of the first and last authors of each reference by databases that store the probability of a first and last name being carried by an author of color. By this measure (and excluding self-citations), our references contain 12.82% author of color (first)/author of color (last), 13.87% white author/author of color, 9.51% author of color/white author and 53.80% white author/white author. This method is limited in that (i) names and Florida Voter Data to make the predictions may not be indicative of racial/ethnic identity, and (ii) it cannot account for Indigenous and mixed-race authors or those who may face differential biases due to the ambiguous racialization or ethnicization of their names. We look forward to future work that could help us to better understand how to support equitable practices in science. This statement was created using cleanBib (*Dalejn cleanBib*, 2020/2020).
