# Supplementary material for: Invoking self-related and social thoughts impacts online information sharing
Source: Soc Cogn Affect Neurosci. 2023 Mar 3;18(1):nsad013. doi: 10.1093/scan/nsad013 (PMC10088358; doi:10.1093/scan/nsad013)
Supplement: nsad013_Supp [file nsad013_supp.zip › SI_SCAN_FINAL_cleancorrection.docx]

Supplementary Materials: Invoking Self-Related and Social Thoughts Impacts Online Information Sharing

Scholz, C.^1^, Baek, E.C.^2^, & Falk, E.B.^3^

^1^ University of Amsterdam

^2^ University of California - Los Angeles

^3^ University of Pennsylvania

Supplementary Materials: Invoking Self-Related and Social Thoughts Impacts Online Information Sharing

## Supplementary Methods

## Additional Participant Characteristics

As mentioned in the main manuscript, participants did not have counter-indications for fMRI scanning, meaning they did not have metal in their body, were not currently pregnant or breast feeding, had no history of neurological disorders, were right-handed, and did not take psychoactive medication at the time of screening,

Participants reported relatively high levels of interest (7-point Likert-type scale) in news about physical activity (M = 5.5, SD = 1.15) and healthy living (M = 5.82, SD = 1.04) and enjoyment of conversations (7-point Likert-type scale) about physical activity (M = 5.4, SD = 1.18) and healthy living (M = 5.58, SD = 1.07), the topics of the articles used as stimuli. Further, our participants read 2-3x per month on the New York Times website and caught up on news in general 2-3x per week.

**Detailed Description of Partial Data Exclusion**

In the main manuscript, we have detailed reasons for participant exclusions (i.e. exclusions of entire datasets). We additionally excluded some participants from a subset of analyses. These partial exclusions and resulting sample sizes for specific analyses are detailed below.

### Neuroimaging and Sharing Likelihood Ratings Data Exclusions.

Eight participants were excluded from all neuroimaging analyses because of excessive head motion (N=2), data loss (N=3) and data corruption (N=3). Further, for three participants one run of fMRI data each was excluded from analysis due to excessive head motion. In sum, we analyzed partial or complete neuroimaging and sharing likelihood data from 42 participants.

### Benefit of Sharing Rating and Sharing Text Data Exclusions.

Sharing benefit ratings from the post-scan task were not collected for one participant. Consequently, 50 participants were included in analyses of text data and 49 in the analysis of sharing benefit rating data.

## Task Instructions

Table S1 prints instructions given to participants in the scan and post-scan tasks to implement the experimental manipulation. In addition, Table S1 shows examples of texts written by participants in the post-scan task for each condition. Texts represent what participants would have said or written to another participant in order to share the a given article with them while following the assigned sharing goal.

Table S1

Instructions and Sharing Texts Written by Participants per Within-Subject Condition for one Original Article

| Conditions | Instructions | Example Sharing Text |
| --- | --- | --- |
| Original Article | – | Finding Your Ideal Running Form: Many experts say runners should be taught the best form, but new research suggests that runners often improve their form just by running more. |
| Describe Yourself | When your goal is to describe yourself, use the article to tell another study participant something about yourself. For instance, you could describe how your interests, experiences, or personality traits relate to the article or tell a story about something you have done or are planning to do in relation to the article topic. You may also express that the article does not relate to you and why. | As a runner I am constantly trying to figure out whats the best most effective way to run and this is definitely a helpful approach |
| Help Somebody | When your goal is to help somebody, use the article to help another study participant. For instance, you could describe how the information in the article could help another study participant solve a problem or why it might be interesting. You may also give somebody the tip to avoid something mentioned in the article and why. | Don’t spend all that money and time on a personal trainer to ‘learn’ how to run! You should read this article that basically says running frequently itself will help your form. |
| Spread Information | When your goal is to spread information, relay information provided in the article to another study participant in your own words without evaluating or changing it. For instance, tell another study participant what the main topic of the article is and what is said about the topic. | New studies have found that runners improve their running form by experience and that teaching runners an ideal form may not be the best way to facilitate runners improving their form |

## fMRI Data Acquisition

For each scan task, we collected two runs of functional images (repetition time (TR) = 1,000 ms, echo time (TE) = 32 ms, flip angle = 60 degrees, −30 degrees tilt relative to the anterior commissure–posterior commissure (ACPC) line, 56 axial slices, field of view = 208 mm, slice thickness = 2.5 mm; voxel size = 2.5 × 2.5 × 2.5 mm, multiband acceleration factor = 4). In addition, we collected high-resolution, T1-weighted, structural images (magnetization-prepared rapid-acquisition gradient echo, inversion time (TI) = 1.100 ms, 160 slices, voxel size = 0.9 × 0.9 × 1 mm), as well as T2-weighted images (TR = 3200 ms, slice thickness = 1 mm, axial slices = 176, voxel size = 1 x 1 x 1 mm) for each participant. T1- and T2-weighted images were used together with the functional images for a two-stage co-registration procedure (see below). Data collection began with a 7.5 s delay in each run to allow the scanner signal to stabilize.

## Pre-Test

We conducted a pre-test of the experimental manipulations discussed in the main manuscript. The goal of the pre-test was to confirm that participants would understand the task, to qualitatively assess the face validity of the task responses, and to explore the potential experimental manipulation. Accordingly, we recruited a small sample online through Amazon’s Mechanical Turk platform and used a small number of trials per participant. The pre-test was not powered for key hypothesis tests.

Fourty-six pre-test participants provided at least one set of texts and ratings in response to an article with condition frame. Of those, three participants were excluded due to non-responsiveness (obscene, unrelated language, n=1, texts consisting entirely of one letter, n=1, copy pasting the same short sentence, n=1). The 43 remaining participants were aged between 23 and 66 (M34.38, SD=10.32). Seventeen participants self-identified as female, 22 as male and none chose an "other" category. We included partial responses (one out of three possible responses per participant) for four participants in the analysis. For those participants, age and gender information is unavailable.

Pre-test participants completed an online survey hosted on Qualtrics where they were presented with three trials of a task that was very similar to the one presented to main study participants. Per trial, pre-test participants saw the abstract and title of a New York Times article (randomly chosen from the sample that was also used in the main study). Each article was attached to a condition prompt equal to those used in the main study (“Describe Yourself”, “Help Somebody”, or “Spread Information”) and each participant completed one trial per condition. After seeing the article and prompt, participants were asked to “Write a short Facebook message about this article with the goal to [CONDITION-SPECIFIC PROMPT] (min. 100 characters)”. Afterwards, pre-test participants rated each message they wrote using three Likert-type scales (1: strongly disagree – 7: strongly agree) similar to those used as manipulation checks in the main study: “The message I wrote reveals something about me.”, “The message I wrote could be helpful to somebody.”, and “The message I wrote describes what is said in the article.” Key differences between the pre-test and the main study include the number of trials per participant (one per condition in the pre-test, eight per condition in the post-scan task of the main study) and the fact that pre-test instructions explicitly mentioned Facebook as a platform to share written messages and articles on while we asked participants in the main study to share with one other study participant.

We used pre-test data to replicate the two manipulation checks that we reported for the main study. Specifically, we used the LIWC dictionary to score sharing texts written in the pre-test and we used the three self-reports regarding participants perception of how successfully each message implemented each of the three sharing motives to examine pre-registered Hypothesis 1.1. For details on these procedures please consult the main manuscript. Results based on pre-test data are presented together with the corresponding results based on data from the main study in the Supplementary Materials.

## Supplementary Analyses and Results

**Manipulation Check Pre-Test**

Reproducing procedures from the main manuscript, we examined whether pre-test participants perceived themselves to be successful in distinguishing between conditions. Confirming Hypothesis 1.1 and replicating results from the main study (Table 1), we found that pre-test participants perceived their messages to be more successful in fulfilling “Describe Yourself” and “Help Somebody” goals when each goal was assigned by condition instructions than when other goals were assigned. Effects regarding the perceived implementation of “Spread Information” goals were not significant in the pre-test due to the small sample size, but directionally confirm main study findings (see Table S2 for pre-test results).

Table S2:

Manipulation Check Results Based on Pre-Test Data. For ‘Help Somebody’ and ‘Describe Yourself’ goals, participants reported that their sharing texts achieved a given goal more effectively when it was assigned via condition prompts than when it was not. This table shows beta estimates and 95% CIs derived using multi-level models regressing self-reported goal fulfillment for each goal (rows) on condition factors, such that column condition > row condition.

| Conditions | Help Somebody | Describe Yourself | Spread Information |
| --- | --- | --- | --- |
| Help Somebody | — | 0.71, [ 0.20;  1.21] | 0.89, [ 0.39;  1.39] |
| Describe Yourself | 1.49, [ 0.78;  2.20] | — | 2.05, [ 1.34;  2.76] |
| Spread Information | 0.38, [-0.11;  0.86] | 0.37, [-0.12;  0.85] | — |

## Condition Effects on How Content is Shared

As discussed in the main manuscript, we used an exploratory analysis of the language written by participants in the post-scan task to further examine the claim that study participants appropriately followed study instructions. We used the LIWC 2007 dictionary (Pennebaker et al., 2007) to score all sharing texts which participants composed as messages to be sent to the receivers of shared content while keeping in mind specific, manipulated sharing goals. The same procedures were applied to pre-test data. Figure S1 shows condition differences in how frequently participants used words from different LIWC categories and Table S3 provides results from the corresponding multi-level models, regressing the extent to which a text contained words from a LIWC category on condition factors. Confirming that participants followed study instructions, we found that participants used more self-related language (LIWC category: “i”) in the “Describe Yourself” compared to both other conditions in both the pre-test and main study. For instance, while sharing an article about people who lie about their results in running races in the “Describe Yourself” condition, one main study participant wrote: “I don’t like exaggerating my exercise results. If I mess with that information I don’t know how I’ve actually improved.” In contrast, across both the pre-test and main study, social language (LIWC category: “social”) was used most often in the “Help Somebody” condition to link articles to other people or give advice such as: “Hey! Maybe ask your doctor about your supplement regime. I don’t think everything Dr. Oz says is good for you….” Finally, only in the main study, in the “Spread Information” condition, participants used more health-related language (LIWC category: “health”) while summarizing the content of health-news articles objectively in their own words. This pattern was directionally present, but not significant in the pre-test data.


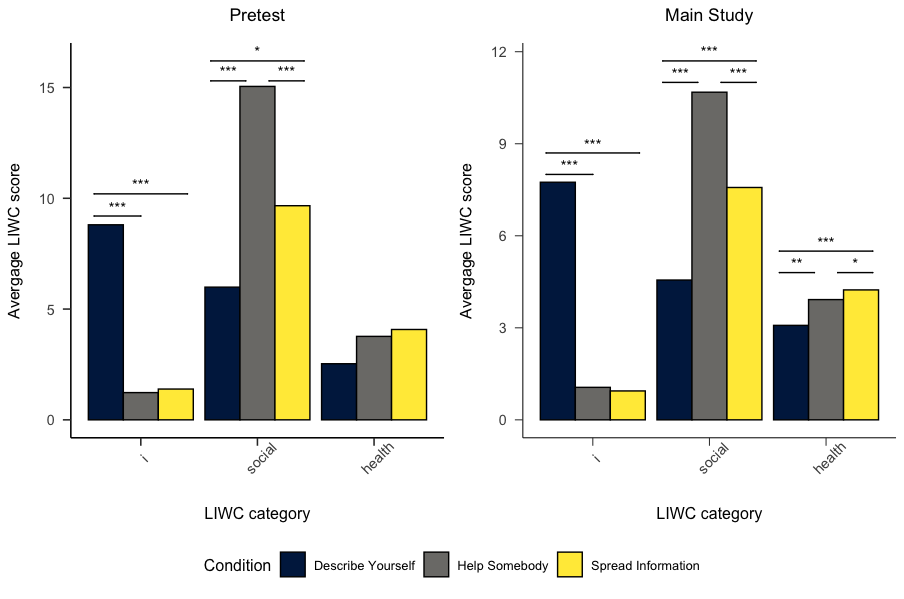


*Figure* S*1.*  Condition Effects on Communicator Sharing Texts in the Pretest and Main Study. Significance tests are derived from multi-level regression models regressing LIWC-scores on dummy variables representing conditions, including random intercepts for participants and articles. Full model results are presented in Supplementary Table 3. *** p < .001, ** p < .01, * p < .05

Table S3:

Condition effects on word category use in the pre-test and main study

|  |  | Pre-Test | | | | | Main Study | | | | |
| --- | --- | --- | --- | --- | --- | --- | --- | --- | --- | --- | --- |
| LIWC category | Contrast | B | se | p | 2.5% | 97.5 % | B | se | p | 2.5% | 97.5 % |
| i | Describe> Spread | 7.40 | 0.76 | < .001 | 5.91 | 8.89 | 6.85 | 0.24 | < .001 | 6.39 | 7.31 |
| i | Help> Spread | -0.20 | 0.76 | 0.795 | -1.69 | 1.29 | 0.16 | 0.24 | 0.493 | -0.30 | 0.63 |
| i | Describe> Help | 7.60 | 0.77 | < .001 | 6.10 | 9.09 | 6.69 | 0.24 | < .001 | 6.22 | 7.15 |
| social | Describe> Spread | -3.81 | 1.53 | 0.017 | -6.83 | -0.83 | -2.98 | 0.39 | < .001 | -3.75 | -2.21 |
| social | Help> Spread | 5.14 | 1.52 | 0.001 | 2.14 | 8.11 | 3.01 | 0.40 | < .001 | 2.23 | 3.80 |
| social | Describe> Help | -8.95 | 1.54 | < .001 | -11.9 | -5.95 | -5.99 | 0.40 | < .001 | -6.78 | -5.20 |
| health | Describe> Spread | -1.03 | 0.84 | 0.228 | -2.70 | 0.65 | -1.27 | 0.23 | < .001 | -1.73 | -0.81 |
| health | Help> Spread | 0.09 | 0.83 | 0.910 | -1.55 | 1.74 | -0.66 | 0.24 | 0.006 | -1.13 | -0.19 |
| health | Describe> Help | -1.13 | 0.85 | 0.190 | -2.77 | 0.52 | -0.61 | 0.24 | 0.011 | -1.08 | -0.14 |

## Effects of Sharing Goals on Brain Activity within Non-Overlapping ROIs

In the main manuscript, we report condition effects on activity within three regions of interest (ROIs). These three ROI masks overlap significantly, raising the question to which extent each cognitive process represented by these ROIs is uniquely affected by the manipulation. To answer this question, we created three additional ROI masks relying on human curated rather than automated meta-analyses and large-scale studies of the cognitions of interest. Specifically, we relied on meta-analyses of self-related processing (Murray et al., 2012) and value-related processing (Bartra et al., 2013) and a large-scale study of mentalizing (Dufour et al., 2013). In a next step, we removed any voxels from each ROI that were also included in one or both of the other ROIs to create non-overlapping masks.

Note that we chose not to use the pre-registered Neurosynth ROIs which are used in the main manuscript as the basis for this exploratory analysis. This is because even after removing physical overlap from these masks, correlation between activity extracted from these ROIs was not reduced significantly.

We then tested whether communicator sharing goals causally affected brain activity in these alternative, non-overlapping ROI masks as operationalizations of self-related, social and value-related processing. Replicating results reported in the main manuscript and in line with pre-registered hypotheses (see H2.1 in https://osf.io/n9vpz), we found increased activity in the self-related ($M=0.00031$, 95% CI $\left[ 0.00003,0.00059 \right]$, $t\left( 41 \right)=2.24$, $p=.031$), mentalizing ($M=0.00209$, 95% CI $\left[ 0.00025,0.00393 \right]$, $t\left( 41 \right)=2.30$, $p=.027$), and value-related ($M=0.00057$, 95% CI $\left[ 0.00011,0.00104 \right]$, $t\left( 41 \right)=2.52$, $p=.016$) ROIs in the “Describe Yourself” compared to the “Spread Information” condition. The “Help Somebody” condition also yielded greater activity in the self-related processing ($M=0.00023$, 95% CI $\left[ 0.00002,0.00044 \right]$, $t\left( 41 \right)=2.20$, $p=.033$) and (marginally) mentalizing ($M=0.00165$, 95% CI $\left[ -0.00004,0.00335 \right]$, $t\left( 41 \right)=1.97$, $p=.056$) ROIs, but not in the value ROI, compared to the “Spread Information” condition. There were no significant differences in activity when comparing the “Describe Yourself” and “Help Somebody” conditions (see RQ2.1c in https://osf.io/n9vpz; Figure S2).


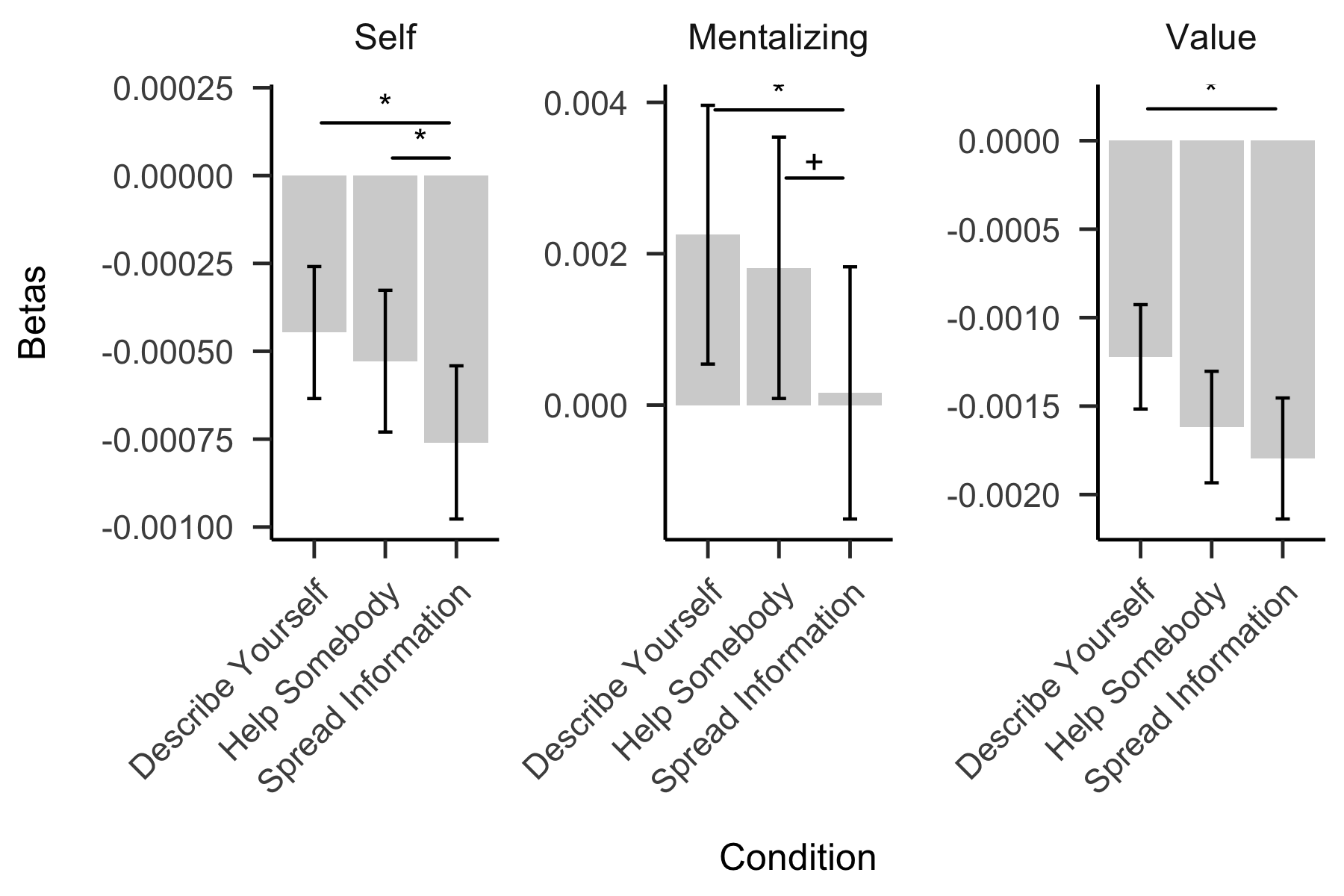


*Figure* S*2.*  Condition Effects on Communication Brain Responses per Condition (each compared to rest) within Non-Overlapping ROI masks. Error bars represent standard errors. Significance indicators are based on one-sample t-tests evaluating average differences in brain activity between condition pairs (see detailed statistics in the main text). * = p < .05

**Condition Effects on Brain Activity Outside the A Priori ROIs**

In the main manuscript, we report analyses highlighting condition effects on activity within our a priori ROIs. Results from two additional analysis approaches, detailed below, further suggest that there was no evidence of widespread condition effects on activity outside of these ROIs.

First, we explored condition effects on activity in the “negative” ROI, which includes the whole brain except voxels included in our three a priori ROIs. Activity in this “negative” ROI was not increased in response to the “Describe Yourself” condition (vs. “Spread Information”; ($M=0.039$, 95% CI $\left[ -0.016,0.095 \right]$, $t\left( 41 \right)=1.43$, $p=.161$) or the “Help Somebody” condition (vs. “Spread Information”; $M=0.004$, 95% CI $\left[ -0.046,0.053 \right]$, $t\left( 41 \right)=0.15$, $p=.884$).

Second, we conducted three pre-registered second-level analyses pooling estimates of brain activity at the individual level across the entire sample of participants using Statistical Parametric Mapping (SPM) software (SPM12, Wellcome Department of Cognitive Neurology, Institute of Neurology, London, UK). Specifically, we computed one-sample t-tests at the second-level for each of the three main contrasts: “Describe Yourself” > “Spread Inforamtion,” “Help Somebody” > “Spread Information,” “Describe Yourself” > “Help Somebody.” Whole brain tables (Tables S4-S6) were created using AtlasReader (Notter et al., 2019). Whole brain maps (Figures S3-S5) were created using the Human Connectome Project Workbench (Markus et al., 2011). No clusters survived FWE correction for multiple comparisons for any of the contrasts. Given the exploratory nature of this analysis, we provide maps and corresponding tables describing clusters in which activity surpasses an uncorrected threshold of p < .001 for each contrast.


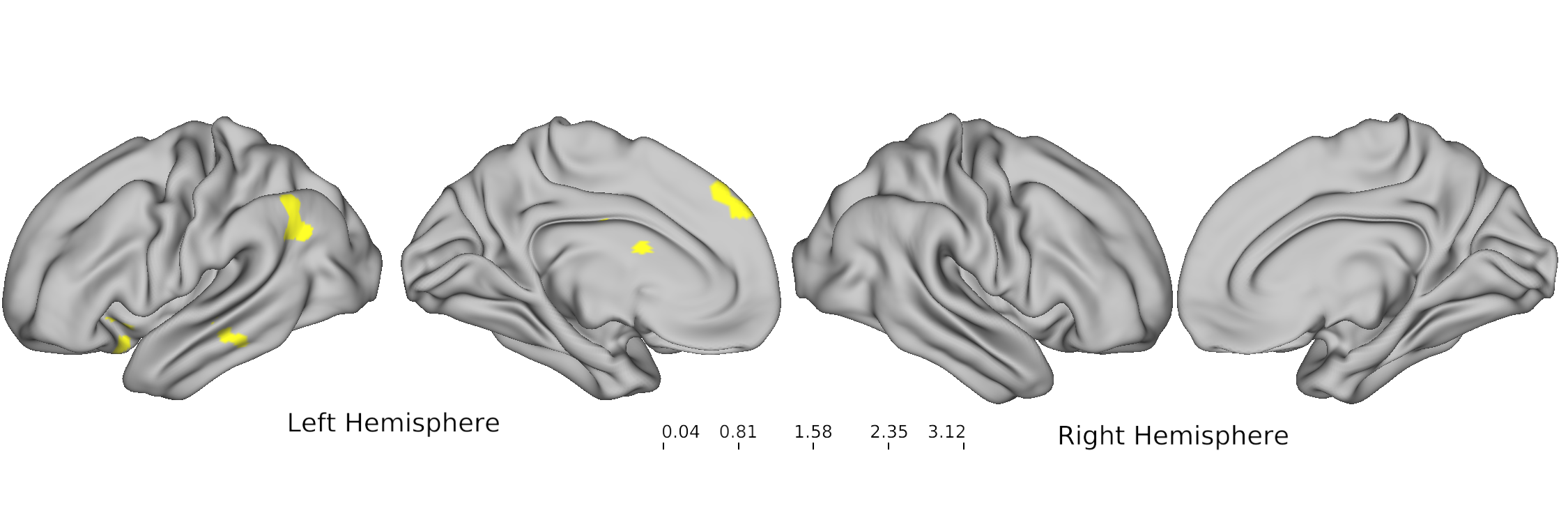


*Figure* S*3.*  Whole brain map “Describe Yourself” > “Spread Information,” uncorrected p < .001

Table S4:

Whole brain table “Describe Yourself” > “Spread Information,” uncorrected p < .001

| x | y | z | Cluster Mean | Volume in mm | aal |
| --- | --- | --- | --- | --- | --- |
| 0.56 | -18.10 | 29.50 | 3.84 | 2,390.92 | 51.92% no_label; 33.97% Cingulate_Mid_R; 14.10% Cingulate_Mid_L |
| 37.70 | -62.67 | 42.00 | 3.48 | 1,915.81 | 56.00% Angular_R; 36.00% Parietal_Inf_R; 7.20% no_label |
| 50.08 | -30.48 | -10.50 | 3.52 | 720.34 | 89.36% Temporal_Mid_R; 10.64% no_label |
| 30.28 | 36.37 | -8.00 | 3.55 | 705.02 | 41.30% OFCant_R; 41.30% Frontal_Inf_Orb_2_R; 13.04% no_label |
| -29.15 | 43.80 | -10.50 | 3.54 | 291.20 | 52.63% Frontal_Mid_2_L; 26.32% OFCant_L; 15.79% Frontal_Sup_2_L; 5.26% Frontal_Inf_Orb_2_L |
| 42.66 | 23.99 | 39.50 | 3.42 | 260.55 | 100.00% Frontal_Mid_2_R |
| -46.48 | -37.91 | 7.00 | 3.52 | 260.55 | 41.18% Temporal_Mid_L; 41.18% Temporal_Sup_L; 17.65% no_label |
| -21.72 | -30.48 | -0.50 | 3.69 | 245.22 | 68.75% Hippocampus_L; 25.00% no_label; 6.25% Thalamus_L |
| 59.99 | -62.67 | 39.50 | 3.38 | 137.94 | 77.78% no_label; 22.22% Angular_R |
| -48.96 | -42.86 | -35.50 | 3.61 | 122.61 | 87.50% Cerebelum_Crus1_L; 12.50% Cerebelum_Crus2_L |
| 45.13 | 36.37 | 37.00 | 3.37 | 76.63 | 100.00% Frontal_Mid_2_R |
| -11.82 | -72.58 | 39.50 | 3.38 | 76.63 | 80.00% Precuneus_L; 20.00% Cuneus_L |


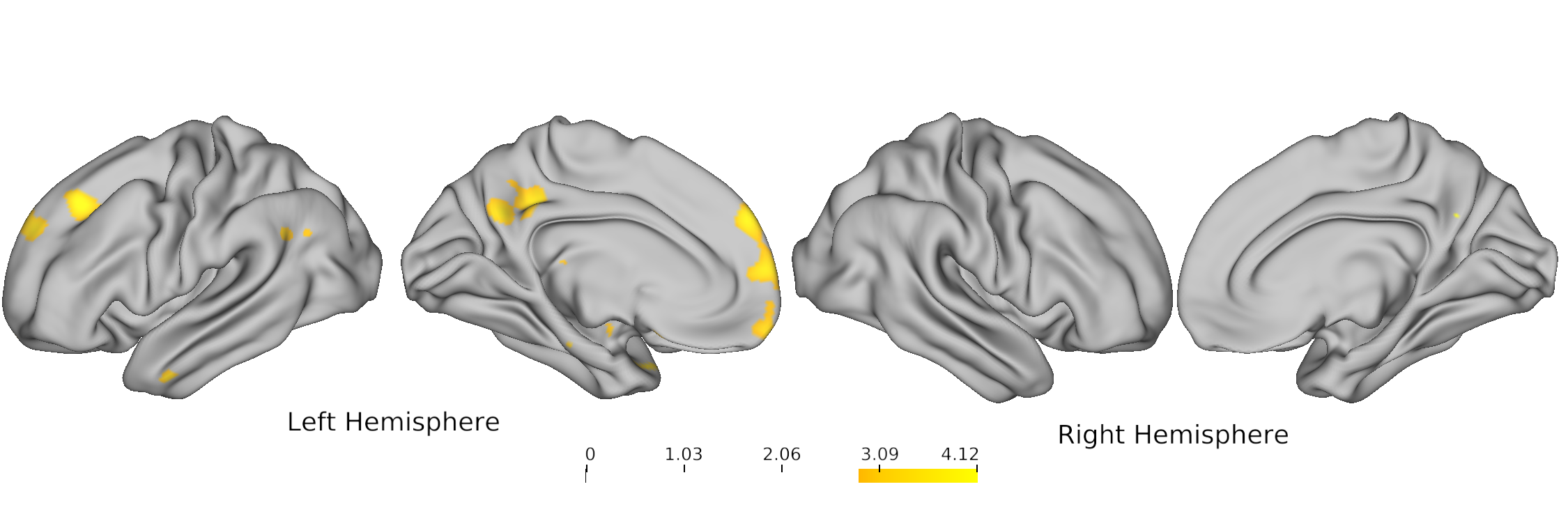


*Figure* S*4.*  Whole brain map “Help Somebody” > “Spread Information,” uncorrected p < .001

Table S5:

Whole brain table “Help Somebody” > “Spread Information,” uncorrected p < .001

| x | y | z | Cluster Mean | Volume in mm | aal |
| --- | --- | --- | --- | --- | --- |
| -24.20 | 33.89 | 49.50 | 3.71 | 4,214.77 | 61.09% Frontal_Sup_2_L; 32.73% Frontal_Mid_2_L; 6.18% no_label |
| 27.80 | -87.43 | -38.00 | 3.70 | 2,023.09 | 71.21% Cerebelum_Crus2_R; 21.97% no_label; 6.82% Cerebelum_Crus1_R |
| -4.39 | 63.60 | 14.50 | 3.55 | 827.63 | 94.44% Frontal_Sup_Medial_L |
| -4.39 | 51.22 | 39.50 | 3.53 | 643.71 | 100.00% Frontal_Sup_Medial_L |
| 0.56 | 11.61 | -25.50 | 3.72 | 521.10 | 94.12% no_label; 5.88% Rectus_R |
| -4.39 | -60.20 | 27.00 | 3.45 | 459.79 | 60.00% Precuneus_L; 23.33% Precuneus_R; 13.33% Cingulate_Post_L |
| -9.34 | -45.34 | 34.50 | 3.48 | 444.47 | 86.21% Cingulate_Mid_L; 13.79% Cingulate_Post_L |
| -9.34 | 9.13 | 19.50 | 3.65 | 413.81 | 66.67% Caudate_L; 33.33% no_label |
| 32.75 | 21.51 | 7.00 | -3.48 | 337.18 | 86.36% Insula_R; 13.64% no_label |
| -36.58 | 14.08 | -25.50 | 3.59 | 321.86 | 100.00% Temporal_Pole_Sup_L |
| 42.66 | 43.80 | 17.00 | -3.48 | 291.20 | 100.00% Frontal_Mid_2_R |
| 3.04 | -57.72 | -55.50 | 3.53 | 275.88 | 61.11% Cerebelum_9_R; 38.89% no_label |
| -56.38 | 1.70 | -28.00 | 3.44 | 260.55 | 76.47% Temporal_Mid_L; 23.53% Temporal_Pole_Mid_L |
| -14.29 | 51.22 | 37.00 | 3.46 | 245.22 | 87.50% Frontal_Sup_2_L; 12.50% Frontal_Sup_Medial_L |
| 10.47 | -47.82 | -43.00 | 3.52 | 229.90 | 100.00% Cerebelum_9_R |
| -46.48 | 41.32 | 14.50 | -3.59 | 229.90 | 100.00% Frontal_Inf_Tri_L |
| 10.47 | 14.08 | 49.50 | -3.57 | 199.24 | 69.23% Supp_Motor_Area_R; 30.77% Frontal_Sup_2_R |
| 42.66 | 38.84 | 27.00 | -3.40 | 183.92 | 66.67% Frontal_Inf_Tri_R; 33.33% Frontal_Mid_2_R |
| -48.96 | 46.27 | -15.50 | 3.62 | 153.26 | 80.00% no_label; 20.00% OFClat_L |
| -24.20 | 66.08 | -8.00 | 3.42 | 137.94 | 44.44% Frontal_Sup_2_L; 33.33% no_label; 22.22% Frontal_Mid_2_L |
| -4.39 | 63.60 | -15.50 | 3.40 | 107.28 | 57.14% Rectus_L; 42.86% Frontal_Med_Orb_L |
| -14.29 | -10.68 | 27.00 | 3.49 | 91.96 | 100.00% no_label |
| -68.76 | -30.48 | -13.00 | 3.49 | 91.96 | 100.00% Temporal_Mid_L |


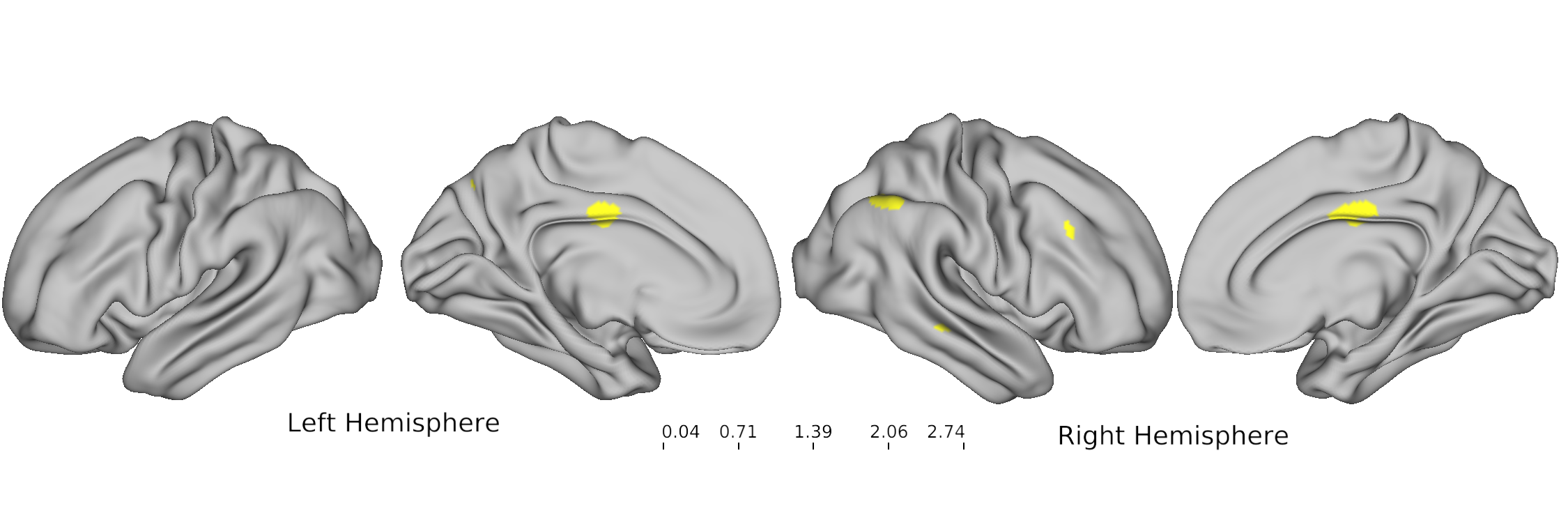


*Figure* S*5.*  Whole brain map “Describe Yourself” > “Help Somebody,” uncorrected p < .001

Table S6:

Whole brain table “Describe Yourself” > “Help Somebody,” uncorrected p < .001

| x | y | z | Cluster Mean | Volume in mm | aal |
| --- | --- | --- | --- | --- | --- |
| 0.56 | -18.10 | 29.50 | 3.84 | 2,390.92 | 51.92% no_label; 33.97% Cingulate_Mid_R; 14.10% Cingulate_Mid_L |
| 37.70 | -62.67 | 42.00 | 3.48 | 1,915.81 | 56.00% Angular_R; 36.00% Parietal_Inf_R; 7.20% no_label |
| 50.08 | -30.48 | -10.50 | 3.52 | 720.34 | 89.36% Temporal_Mid_R; 10.64% no_label |
| 30.28 | 36.37 | -8.00 | 3.55 | 705.02 | 41.30% OFCant_R; 41.30% Frontal_Inf_Orb_2_R; 13.04% no_label |
| -29.15 | 43.80 | -10.50 | 3.54 | 291.20 | 52.63% Frontal_Mid_2_L; 26.32% OFCant_L; 15.79% Frontal_Sup_2_L; 5.26% Frontal_Inf_Orb_2_L |
| 42.66 | 23.99 | 39.50 | 3.42 | 260.55 | 100.00% Frontal_Mid_2_R |
| -46.48 | -37.91 | 7.00 | 3.52 | 260.55 | 41.18% Temporal_Mid_L; 41.18% Temporal_Sup_L; 17.65% no_label |
| -21.72 | -30.48 | -0.50 | 3.69 | 245.22 | 68.75% Hippocampus_L; 25.00% no_label; 6.25% Thalamus_L |
| 59.99 | -62.67 | 39.50 | 3.38 | 137.94 | 77.78% no_label; 22.22% Angular_R |
| -48.96 | -42.86 | -35.50 | 3.61 | 122.61 | 87.50% Cerebelum_Crus1_L; 12.50% Cerebelum_Crus2_L |
| 45.13 | 36.37 | 37.00 | 3.37 | 76.63 | 100.00% Frontal_Mid_2_R |
| -11.82 | -72.58 | 39.50 | 3.38 | 76.63 | 80.00% Precuneus_L; 20.00% Cuneus_L |

## Pre-Registered Analyses: Causal Effects of Sharing Goals on Sharing Likelihood

Our pre-registered analysis plan included the use of one-sample t-tests to examine condition-differences in self-reported sharing likelihood and perceived benefits of sharing. While we report the results of multi-level models in the main manuscript, the results of the pre-registered analysis are reported in Table S7 below. At this aggregated level of analysis, which has less statistical power than the multi-level models, one of the results (effect of Help Somebody vs. Spread Information on sharing likelihood) presented as significant in the main paper using a multi-level model does not reach significance at an alpha level of .05 using the aggregated data. All other findings produce equivalent substantive conclusions. Thus, we interpret the inconsistent finding with more caution in the discussion section. Results for the perceived benefit of sharing ratings are fully consistent with those reported in the main manuscript. “Spread Information” instructions and “Help Somebody” instructions lead to higher perceived benefits of sharing than “Describe Yourself” instructions, and “Help Somebody” instructions outperformed “Spread Information” instructions.

Table S7:

Paired t-tests comparing sharing likelihood and perceived benefit of sharing ratings between conditions

| Rating | Contrasts | T-tests |
| --- | --- | --- |
| Sharing Likelihood | Describe Yourself vs. Spread Information | MΔ = -0.080, 95% CI[-0.240, 0.081], t(49) = -0.99, p = .325 |
| Sharing Likelihood | Help Somebody vs. Spread Information | MΔ = 0.115, 95% CI [-0.020, 0.249], t(49) = 1.71, p = .093 |
| Sharing Likelihood | Describe Yourself vs. Help Somebody | MΔ = -0.194, 95% CI [-0.341, -0.048], t(49) = -2.66, p = .010 |
| Sharing Benefit | Describe Yourself vs. Spread Information | MΔ = -0.193, 95% CI [-0.347, -0.039], t(48) = -2.52, p = .015 |
| Sharing Benefit | Help Somebody vs. Spread Information | MΔ = 0.191, 95% CI [0.044, 0.338], t(48) = 2.61, p = .012 |
| Sharing Benefit | Describe Yourself vs. Help Somebody | MΔ = -0.384, 95% CI [-0.532, -0.235], t(48) = -5.18, p < .001 |

We chose to deviate from our analysis plan and to report multi-level models in the main manuscript, because we realized that multi-level models were more appropriate given the repeated measures in our data. In addition, we note that at an alpha-level of 0.05 and with an effect size of Cohen’s d = 0.14 for the comparison of sharing likelihood between the “Describe Yourself” and “Spread Information” condition, the pre-registered test reaches only 25.2% power. However, we argue that small effect sizes are practically important in this work given the large number of individual shares that occur in real-life online environments on a daily basis. Even small, consistent changes in the percentage of readers who decide to share a piece of information can have meaningful effects on both the sharers and those who receive shared content.

## Indirect Condition Effects on Brain Activity in the Value-Related ROI

Although we found hypothesized positive direct effects of the “Help Somebody” condition (relative to “Spread Information”) on brain activity in the self-related and social ROIs, we did not find similar direct effects on activity in the value-related ROI. Previous work has theorized that value-related activity acts as a final common pathway in the brain where other sources of value (e.g. perceptions of self-related values like the ability to self-promote and social values like opportunities to bond) are integrated into a final judgment of the value of information sharing (Falk & Scholz, 2018). Consequently, we tested whether our manipulation had an indirect effect on value-related activity through brain activity in the other two ROIs. For these exploratory multi-level regression models, we estimated a first-level model of the brain data including separate regressors for each trial to extract brain activity in response to each article (i.e. a beta series model to allow us to extract one value per article, per participant). Each regressor included the entire period within the trial during which the article was visible on the screen. We then conducted exploratory multi-level regression analysis using indirect.mlm in R (Page-Gould & Sharples, 2016) to test indirect effects of condition on activity in the value-related ROI, mediated by activity in the other two ROIs.

Both indirect effects were positive and significant as shown in Table S8.

Table S8:

Multi-Level Models Regressing Value-Related Brain Activity on Condition (Help Somebody > Spread Information), Mediators: self-related and social ROI activity; B [Bootstrapped 95%-CI with 1000 bootstrap samples]

| Mediator ROI | a-path | b-path | c’-path | Indirect Effect | Total Effect |
| --- | --- | --- | --- | --- | --- |
| self | 0.14 [0.032, 0.248] | 0.886 [0.861, 0.912] | -0.044 [-0.092, 0.007] | 0.124 [0.029, 0.221] | 0.08 [-0.024, 0.188] |
| social | 0.149 [0.047, 0.262] | 0.845 [0.819, 0.875] | -0.046 [-0.104, 0.015] | 0.126 [0.038, 0.219] | 0.079 [-0.034, 0.2] |

##

## Indirect Condition Effects on Sharing Intentions

Contrary to our hypotheses, self-report ratings indicated that the “Describe Yourself” condition decreased perceived benefits and intentions to share relative to the “Spread Information” condition. However, the observed effects on brain activity supported our hypotheses, such that “Describe Yourself” elicited greater activity in hypothesized brain regions tracking self-related, social and value-related processing. To investigate these findings further, we ran an exploratory trial-wise, multi-level regression model to test whether the main effect of condition (“Describe Yourself” > “Spread Information”) on sharing ratings was (partially) mediated by brain activity in our three ROIs. For this analysis, trial-wise brain activity was standardized within participant.

As shown in Table S9, we found that the direct negative effect of condition (“Describe Yourself” > “Spread Information”) on sharing likelihood rating (c’-path) was partially mediated by a positive indirect effect through activity in all three ROIs, such that the “Describe Yourself” relative to the “Spread Information” condition led to higher ROI activation, and this increased ROI activity was positively associated with sharing likelihood ratings. In other words, we find two mechanisms that explain the association between “Describe Yourself” instructions (compared to control) and sharing likelihood ratings: a direct effect indicating lower sharing likelihood ratings for “Describe Yourself” and an additional indirect effect where “Describe Yourself” instructions have a positive effect on sharing likelihood ratings via increases in the activation of specific ROIs.

Table S9:

Multi-Level Models Regressing Sharing Likelihood Ratings on Condition ((“Describe Yourself” > “Spread Information”), Mediated by self-related, mentalizing, and value-related ROI activity, respectively; B [Bootstrapped 95%-CI with 1000 bootstrapped samples]

| ROI | a-path | b-path | c’-path | Indirect Effect | Total Effect |
| --- | --- | --- | --- | --- | --- |
| value | 0.128 [0.014, 0.238] | 0.155 [0.096, 0.218] | -0.12 [-0.256, -0.001] | 0.02 [0.002, 0.046] | -0.1 [-0.232, 0.018] |
| self | 0.18 [0.076, 0.291] | 0.118 [0.052, 0.174] | -0.12 [-0.259, -0.002] | 0.021 [0.007, 0.049] | -0.099 [-0.234, 0.02] |
| mentalizing | 0.172 [0.055, 0.286] | 0.094 [0.038, 0.156] | -0.113 [-0.243, -0.003] | 0.016 [0.002, 0.038] | -0.097 [-0.225, 0.017] |

# References

Bartra, O., McGuire, J. T., & Kable, J. W. (2013). The valuation system: A coordinate-based meta-analysis of BOLD fMRI experiments examining neural correlates of subjective value. *NeuroImage*, *76*, 412–427. https://doi.org/10.1016/j.neuroimage.2013.02.063

Dufour, N., Redcay, E., Young, L., Mavros, P. L., Moran, J. M., Triantafyllou, C., Gabrieli, J. D. E., & Saxe, R. (2013). Similar brain activation during false belief tasks in a large sample of adults with and without autism. *PLoS ONE*, *8*(9), e75468. https://doi.org/10.1371/journal.pone.0075468

Falk, E. B., & Scholz, C. (2018). Persuasion, Influence, and Value: Perspectives from Communication and Social Neuroscience. *Annual Review of Psychology*, *69*(1), 329–356. https://doi.org/10.1146/annurev-psych-122216-011821

Markus, D. S., Harwell, J., Olsen, T., Hodge, M., Glasser, M. F., Prior, F., Jenkinson, M., Laumann, T., Curtiss, S. W., & Van Essen, D. C. (2011). Informatics and data mining: Tools and strategies for the Human Connectome Project. *Frontiers in Neuroinformatics*, *5*(4).

Murray, R. J., Schaer, M., & Debbané, M. (2012). Degrees of separation: A quantitative neuroimaging meta-analysis investigating self-specificity and shared neural activation between self- and other-reflection. *Neuroscience & Biobehavioral Reviews*, *36*(3), 1043–1059. https://doi.org/10.1016/j.neubiorev.2011.12.013

Notter, M. P., Gale, D., Herholz, P., Markello, R. D., Notter-Biesler, M.-L., & Whitaker, K. (2019). *AtlasReader: A Python package to generate coordinate tables, region labels, and informative figures from statistical MRI images*. *4*(34), 1257. https://doi.org/10.21105/joss.01257

Page-Gould, E., & Sharples, A. (2016). *Indirect.mlm*. http://www.page-gould.com/r/indirectmlm/

Pennebaker, J. W., Chung, C. K., Ireland, M., Gonzales, A., & Booth, R. J. (2007). *The development and psychometric properties of LIWC2007*. http://homepage.psy.utexas.edu/HomePage/faculty/Pennebaker/reprints/LIWC2007_LanguageManual.pdf
